# Structural Elucidation of Irish Ale Bioactive Polar Lipids with Antithrombotic Properties

**DOI:** 10.3390/biom10071075

**Published:** 2020-07-18

**Authors:** Alexandros Tsoupras, Ronan Lordan, Eoin O’Keefe, Katie Shiels, Sushanta Kumar Saha, Ioannis Zabetakis

**Affiliations:** 1 Department of Biological Sciences, University of Limerick, V94 T9PX Limerick, Ireland; Ronan.Lordan@ul.ie (R.L.); Eoin.OKeeffe@ul.ie (E.O.); Ioannis.Zabetakis@ul.ie (I.Z.); 2 Health Research Institute, University of Limerick, V94 T9PX Limerick, Ireland; 3 Bernal Institute, University of Limerick, V94 T9PX Limerick, Ireland; 4 Shannon Applied Biotechnology Centre, Limerick Institute of Technology, Moylish Park, V94 E8YF Limerick, Ireland; Katie.Shiels@lit.ie (K.S.); Sushanta.Saha@lit.ie (S.K.S.)

**Keywords:** beer, CVD, PAF, polar lipids, platelet aggregation, fatty acids, PUFA, anti-inflammatory

## Abstract

The structures of bioactive polar lipids (PLs) of Irish ale with potent antithrombotic and cardioprotective properties were elucidated. Ale PL was fractionated by preparative thin layer chromatography (TLC) into subclasses, and their antithrombotic effect was assessed against human platelet aggregation induced by the pro-inflammatory mediator, platelet-activating factor (PAF). The fatty acid content and the overall structures of ale PL were elucidated by liquid chromatography mass spectrometry (LC-MS). Phosphatidylcholines (PC) and molecules of the sphingomyelin (SM) family exhibited the strongest anti-PAF effects, followed by phosphatidylethanolamines (PE). PC contained higher amounts of omega-3 polyunsaturated fatty acids (n-3 PUFA) and thus the lowest n-6/n-3 ratio. Bioactive diacyl and alkyl-acyl PC and PE molecules bearing n-3 PUFA at their *sn*-2 position, especially docosahexaenoic acid (DHA) and α-linolenic acid (ALA) but mostly oleic acid (OA), were identified in both PC and PE subclasses. Eicosapentaenoic acid (EPA) was present only in bioactive PC molecules and not in PE, explaining the lower anti-PAF effects of PE. Bioactive sphingolipid and glycolipid molecules with reported anti-inflammatory and anti-tumour properties, such as specific ceramides and glucosylcerebrosides with sphingosine, phytosphingosine and dihydrosphingosine bases but also specific monogalactodiglycerides and SM species bearing ALA at their *sn*-2 position, were identified in the SM subclass, providing a rational for its strong bioactivities against the PAF pathway. Further studies are required on the health benefits of bioactive PL from beer and brewery by-products.

## 1. Introduction

Human platelets are critically involved in normal haemostasis, while their abnormal activation is implicated in thrombosis and/or pathological bleeding [1,2,3]. Apart from their role in haemostasis, activated platelets acting together with other cells, including white blood cells, endothelial cells and/or smooth muscle cells, play a crucial part in inflammation and in the onset and progression of related chronic pathologies, which include cardiovascular diseases (CVDs) and cancer [1,2,3]. Notwithstanding the multiple roles that platelets possess, the study of compounds affecting platelet reactivity induced by different inflammatory and thrombotic mediators and platelet agonists implicated in CVD processes is of great importance for the design of future therapies and interventions. Indeed, dietary strategies may also play a significant role [1].

Several food products derived from the fermentation of food-related raw materials have been found to contain ingredients with specific biological activities and health benefits against these disorders, such as antioxidant, antimicrobial, antifungal, anti-inflammatory, antidiabetic and anti-atherosclerotic activities [4,5,6,7,8,9]. Specific beverages produced from fermentation, including alcoholic ones, also contain bioactive ingredients, such as bioactive polar lipids (PLs) with antithrombotic properties against platelet aggregation [10,11,12,13,14]. Amongst such fermented products/beverages containing bioactive PL with highly potent antithrombotic properties, beer is one of these [13,14]. We have previously described that Irish beer extracts rich in bioactive PL have been found to inhibit platelet aggregation induced by the inflammatory and thrombotic mediators, namely platelet-activating factor (PAF) and thrombin [13,14].

PAF is a phospholipid mediator that acts through specific membrane G protein-coupled receptors (GPCR) in several cells, including platelets, namely the PAF-receptor (PAF-R) [15]. Binding of PAF on its receptor activates specific signalling pathways implicated in inflammatory and thrombotic activation of platelets and several other cells, including leucocytes and endothelial cells, during atherosclerotic and atherothrombotic events in CVD and other inflammation-related chronic disorders [2,3,15,16,17]. Several bioactive PLs derived from healthy foods provide health benefits by affecting the PAF-related inflammatory and thrombotic signalling pathways through antagonism for PAF-R and by beneficially affecting PAF metabolism towards homeostatic PAF levels and thus by reducing PAF-related pro-inflammatory cascades [3,18,19]. The structures for several bioactive PL (phospholipids and glycolipids) from a variety of such healthy food-related sources with potent anti-PAF beneficial properties have been elucidated, along with structure–activity relationships for these PL and their antagonistic effects against PAF binding on its PAF-R because of structural homologies to PAF [20,21,22,23,24].

The aim of the present study was to elucidate the structures of the bioactive PL of Irish ale with antithrombotic properties against PAF-induced human platelet aggregation for the first time. In order to achieve such a goal, Irish ale extracts rich in such bioactive PLs [13] were further fractionated by preparative thin layer chromatography (TLC) into their PL subclasses, including phosphatidylcholines (PC), phosphatidylethanolamines (PE) and PL molecules of the sphingomyelin (SM) family. The antithrombotic properties of each PL subclass were assessed against PAF-induced aggregation of human platelets, while the fatty acid content and the overall structures of the bioactive beer PL in each subclass were elucidated by liquid chromatography mass spectrometry (LC-MS) analysis.

## 2. Materials and Methods

### 2.1. Materials and Instrumentation

All glass and plastic consumables, reagents and solvents were of analytical grade and were purchased from Fisher Scientific Ltd. (Dublin, Ireland); 20G safety needles and evacuated sodium citrate S-monovettes for phlebotomy were purchased from Sarstedt Ltd. (Wexford, Ireland). All platelet assay consumables were purchased from Labmedics LLP (Abingdon on Thames, UK). Standard PAF, egg phospholipid extract, and bovine serum albumin (BSA) were purchased from Sigma Aldrich (Wicklow, Ireland). Centrifugations were carried out on an Eppendorf 5702R centrifuge (Eppendorf Ltd., Stevenage, UK). Spectrophotometric analysis was carried out on a Shimadzu UV-1800 spectrophotometer (Kyoto, Japan) using a quartz 1 cm cuvette.

### 2.2. Samples Irish Ale Assessed

The beer samples (n = 6) assessed in this study were obtained from the Munster Brewery facility (Youghal, Co. Cork, Ireland). The beer is an organically produced Irish red ale for commercial sale, under the name “12 Towers” brewed in accordance with organic standards certified by the Irish Organic Association, as previously described [13].

### 2.3. Extraction and Separation of Polar Lipid Extracts from Irish Ale Samples

The extraction of ale total lipid (TL) extracts and their separation to extracts rich in neutral lipids (NLs) and PLs were performed as previously described [13,14]. Briefly, TLs were extracted from beer samples according to the Bligh and Dyer method [25] and further subjected to countercurrent distribution according to Galanos and Kapoulas [26] in order to obtain the extracts rich in PL and NL. Solvents were evaporated from all extracts using flash rotary evaporation under vacuum (Buchi Rotavapor, Mason Technology, Dublin, Ireland), and lipid samples were transferred into small glass vials, where all the remaining solvents were further evaporated under a stream of nitrogen. The acquired TL, NL and PL extracts were weighed, re-dissolved in 1 mL of chloroform–methanol (1:1; *v*/*v*) and stored at −20 °C in a nitrogen atmosphere until further analysis.

### 2.4. Fractionation of Bioactive Irish Ale PL Extracts to PL Subclasses by Preparative TLC

The TLC analysis of the bioactive Irish ale PL extracts into PL subclasses was performed as previously described [14,22,23,24]. Briefly, up to 50 mg of ale PL extracts were applied to the TLC plates. An elution system consisting of chloroform–methanol–water (65:35:6; *v*/*v*/*v*) was utilised for the separation of ale PL. Subsequently, the plates were stained under iodine vapours. Six major bands appeared after the separation of the ale PL into PL subclasses. Following the evaporation of the iodine vapours, the bands were scraped and lipids were extracted from the silica gel according to the Bligh and Dyer method [25]. The chloroform phase was evaporated to dryness under nitrogen, and lipids in each TLC band were weighed, re-dissolved in 1 mL of chloroform–methanol 1:1 (*v*/*v*) and stored at −20 °C in a nitrogen atmosphere until further analysis.

### 2.5. Assessment of Antithrombotic Properties of Beer PL Subclasses Against PAF-Induced Human Platelet Aggregation

The Ethics Committee of the University of Limerick approved the protocol, which was performed in accordance with the Declaration of Helsinki. Healthy donors (n = 6) were made aware that their blood samples were used in our study, and written consent was provided. Blood collection and preparation of human platelet-rich plasma (hPRP) and all platelet aggregation bioassays were carried out on a Chronolog-490 two-channel turbidimetric platelet aggregometer (Havertown, PA, USA), coupled to the accompanying AGGRO/LINK software package as previously described [23,27].

Briefly, the blood samples were collected from each donor by a phlebotomist in sodium citrate anticoagulant and were centrifuged at 194× *g* for 18 min at 24 °C with no brake applied. The supernatant hPRP was then transferred to polypropylene tubes at room temperature for the aggregation bioassays, whereas platelet-poor plasma (PPP) was obtained by further centrifuging the specimens at 1465*× g* for 20 min at 24 °C with no brake applied. hPRP was adjusted to 500,000 platelets/µL if required by the addition of the respective volume of PPP according to the absorbance of the hPRP measured in a spectrophotometer.

Aliquots of standard PAF stock solution in chloroform/methanol (1:1 *v*/*v*) were evaporated under a stream of nitrogen and re-dissolved in BSA (2.5 mg BSA/mL saline) to obtain PAF solutions with final concentrations into the aggregometer cuvette ranging from 0.26 nM to 0.26 μM. The examined TLC-derived bands of ale PL subclasses were also dissolved in BSA (2.5 mg BSA/mL saline).

Then, 250 µL of PRP was added to an aggregometer cuvette at 37 °C with stirring at 1000 rpm. The PRP was calibrated using the PPP as a blank. The maximum-reversible PAF-induced platelet aggregation was determined as 100% aggregation, which was also used as baseline (0% inhibition) in the absence of any lipid sample, by adding appropriate amounts of PAF in the aggregometer cuvette in order to reach specific final concentrations for PAF approximately 0.1–1 nM.

The PAF-induced aggregation of hPRP was calculated first at 0% inhibition of baseline in a cuvette (100% aggregation) in the absence of any lipid sample, whereas after the preincubation of hPRP with several amounts (μg) of the test lipid samples for 2 min (a different cuvette was used for each amount of the lipids tested), the same amount of PAF was added and the reduced aggregation was calculated. Thus, a linear curve at the 20–80% range of the percentage of inhibition against PAF-induced aggregation of hPRP to the concentrations of each lipid sample was deduced. From this curve, the concentration (μg) of the lipid sample that led to 50% of the PAF-induced aggregation of hPRP was calculated as the 50% inhibitory concentration value also known as the IC_50_ value (half-maximal inhibitory concentration) for each sample.

The resulting IC_50_ values were expressed as a mean value of the mass of a specific ale PL subclass (µg) in the aggregometer cuvette ± standard deviation (SD). All experiments were performed several times for each ale PL subclass sample (N = 6) using a different donors’ blood sample for each replicate.

### 2.6. Fatty Acid Content and Structural Elucidation of Ale PL Subclasses by LC-MS Analysis

The most bioactive TLC-derived ale PL subclasses (corresponding to the PC, PE and SM-family subclasses) against the PAF pathway of human platelet aggregation were further analysed by LC-MS as previously described [24] in order to elucidate their overall structures and their fatty acid composition (Free fatty acids; FFA) derived from their saponification.

Briefly, each of these lipid samples (TLC-derived ale PL subclasses corresponding to the PC, PE and SM-family subclasses) was separated into two half parts and dried in a N_2_ stream. The first half of each sample was saponified by adding 1.5 mL of a saponification reagent, (2.5 M KOH: methanol (1:4, *v*/*v*)), which was gently vortexed. The vials were incubated at 72 °C for 15 min prior to the addition of 225 µL of formic acid. Then, 1725 µL of chloroform and 375 µL of Milli-Q water were added and vortexed to separate the two layers. The chloroform layer containing FFA was transferred carefully to amber vials and evaporated to dryness before being stored at −20 °C until LC-MS analysis.

Before LC-MS analysis, all of the dried lipids were reconstituted in 500 µL of methanol–dichloromethane (2:1; *v*/*v*), centrifuged at 13,793*× g* for 6 min (Heraeus Biofuge Stratos, Fisher Scientific Ltd., Dublin, Ireland) prior to filtering through 3 kDa ultra-centrifuge filters (Amicon Ultra 3k, Merck Millipore Ltd., Carrigtwohill, Co. Cork, Ireland). Polar lipid and FFA profiles were obtained from an HPLC (Agilent 1260 series, Agilent Technologies Ireland Ltd., Little Island, Co. Cork, Ireland) equipped with a Q-TOF mass spectrometer (Agilent 6520), and the source type was electrospray ionization (ESI). The column used for the separations was an Agilent C18 Poroshell 120 column (2.7 µm, 3.0 × 150 mm). The composition of the mobile phase (A) was 2 mM ammonium acetate in water and 2 mM ammonium acetate in 95% acetonitrile for the mobile phase (B). Chromatographic separation was performed by gradient elution starting with 60% B for 1 min and then by increasing to 90% B over 2.5 min. Subsequently, 90% B was held for 1.5 min and increased afterward to 100% over 5 min. Then, 100% B was held for 4 min, reducing afterward to 60% B over 0.5 min and held for 1 min until the next run. The mobile phase flow rate was 0.3 mL/min until 5 min elapsed, increasing up to 0.6 mL/min after 10 min and held at this flow rate until the end of the run. The injection volume was 10 μL. The mass spectrometer was operated in negative ionization mode, scanning the lipids from *m*/*z* 50–1100. Drying gas flow rate, temperature and nebuliser pressure were at 5 L min^−1^, 325 °C and 30 psi, respectively. Fragmentor and skimmer voltages were kept at 175 V and 65 V, respectively, and the capillary voltage was 3500 V. In the negative ion mode, the monitoring reference masses used were 1033.988 and 112.9855, respectively.

The assignment of FFA and phospholipid species was based upon a combination of survey, daughter, precursor and neutral loss scans, as previously described [24] The identity of the bioactive PL molecules was verified using the LIPID MAPS: Nature Lipidomics Gateway (www.lipidmaps.org) by using the lowest delta values combined with the results obtained from the LC-MS analysis of the FFA that were produced by their saponification, as previously described [24].

### 2.7. Statistical Analysis

One-way analysis of variance (ANOVA) was used for all comparisons of IC_50_ values against PAF-induced aggregation of human platelets, while Kruskal Wallis nonparametric multiple comparison tests was used for comparisons in the FA composition acquired from the LG-MS analysis. Differences were considered to be statistically significant when the *p* value was less than 0.05 (*p* < 0.05). The data were analysed using a statistical software package (IBM-SPSS statistics 25 for Windows, SPSS Inc., Chicago, IL, USA).

## 3. Results

### 3.1. Separation of Bioactive PL Extracts from Irish Ale into PL Subclasses by TLC and Evaluation of Their Anti-Thrombotic Potency Against the PAF Pathway of Platelet Aggregation

**Figure 1:**
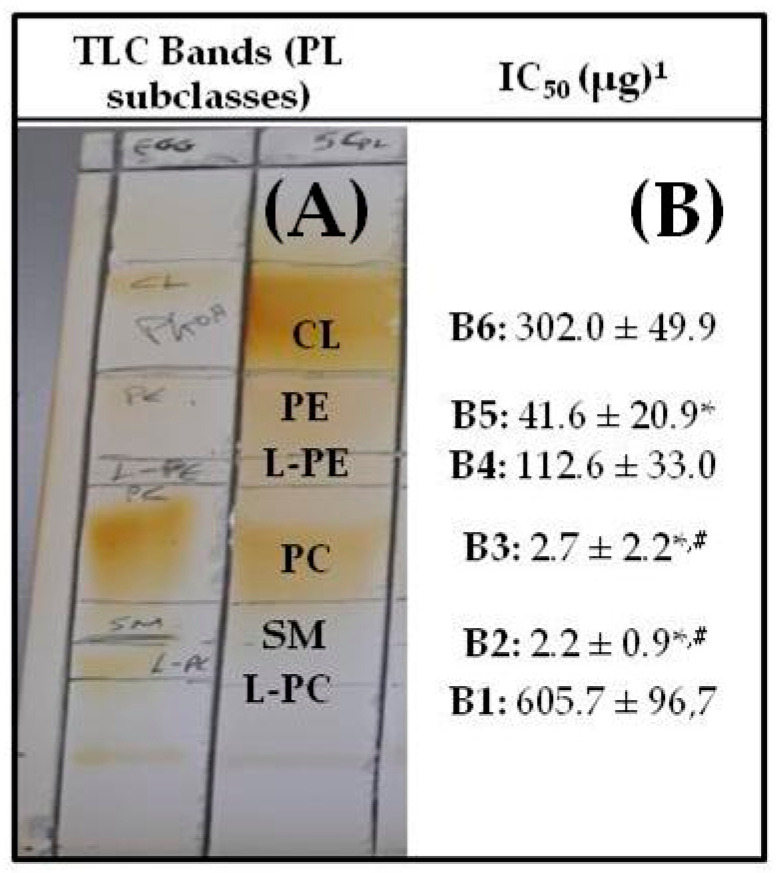
Thin layer chromatography (TLC) analysis of bioactive Irish ale polar lipid (PL) extracts into PL subclasses (**A**), along with the in vitro antithrombotic properties of TLC-derived bands of the PL subclasses against platelet-activating factor (PAF)-induced aggregation of human platelets (**B**). (**A**) For the TLC analysis, a standard mixture of egg phospholipids was co-assessed (left column of the TLC plate) with the Irish ale PLextract (right column of the TLC plate), and relevant bands—PL subclasses—were visualised by I2 vapours for both the standard and sample. (**B**) ^1^ Results are expressed as mean values ± SD of IC_50_ measured as the mass of the ale PL subclass (µg) in the aggregometer cuvette that inhibited 50% of the PAF-induced platelet aggregation in human platelet-rich plasma (hPRP); * statistically significant differences (*p* < 0.05) of the bioactivities of ale PL subclasses of TLC bands 2 (sphingomyelin (SM)), 3 (phosphatidylcholines (PC)) and 5 (phosphatidylethanolamines (PE)) when compared to the other bands 1 (lyso-PC (L-PC)), 4 (lyso-PE (L-PE)) and 6 (egg cardiolipin (CL)). ^#^ Statistically significant differences (*p* < 0.05) of the bioactivities of ale PL subclasses of TLC bands 2 (SM) and 3 (PC) when compared to the band 5 (PE). The results are representative of independent experiments in blood samples of different healthy donors (n = 6) in order to ensure reproducibility. SD: standard deviation; PC: Phosphatidylcholines; PE: Phosphatidylethanolamines; SM: molecules of the sphingomyelin family; L-PC: lyso-phosphatidylcholines; L-PE: lyso-phosphatidylethanolamines; CL: egg cardiolipin.

Bioactive PL extracts of Irish ale were fractionated by TLC into several PL subclasses by preparative TLC analysis, as previously described [14,23,24]. Six major PL subclasses of the bioactive PL from Irish ale were determined in specific TLC bands when compared to specific standards of phospholipid subclasses; TLC bands 1–6 of the Irish ale PL extracts were found to possess similar R_f_ values to those of lyso-PC, polar lipids of the SM family, PC, lyso-PE, PE and cardiolipin (CL) (Figure 1).

The antithrombotic potency for each one of these Irish ale PL subclasses is also shown in Figure 1, where their anti-PAF bioactivities are expressed as IC_50_ values (half-maximal inhibitory concentration) of a PL subclass that led to 50% inhibition of the PAF-induced aggregation of hPRP. It should be noted that the IC_50_ values reflect the antithrombotic potency of each Irish ale PL subclass against the PAF-pathway, since the lower the IC_50_ value for a PL subclass, the higher its anti-PAF potency.

### 3.2. Fatty Acid Composition of the Most Bioactive PL Subclasses, SM, PC and PE from Irish Ale

The fatty acid profile for each one of these bioactive PL subclasses (SM, PC, and PE), obtained from the LC-MS analysis of the saponified PL subclasses of Irish ale PL, are shown in Table 1 (characteristic chromatograms of this analysis are shown in supplementary data, as Figure S1). By using a C18 reverse-phase column in the LC-MS analysis, the separation of the FFA was mostly based on the length of their carbon chain in combination with their degree of unsaturation. By applying quadrupole time-of-flight mass spectrometry (Q-TOF) simultaneously with the HPLC separation for each one of these peaks, unique MS data were obtained for each peak, leading to complete quantification of each fatty acid present in these PL subclasses. The characterization and quantification of these molecules was based on survey scans performed in the negative ion mode between 200 and 1000 *m*/*z* and the acquired *m*/*z* values of the dehydrogenated negative ions [M − H]^−^ for each one of the fatty acid that are also shown in Table 1, as previously described [24].

More specifically, the saturated fatty acids (SFA) were the most dominant fatty acid class in TPL of the most bioactive PL subclasses (SM, PC and PE) from Irish ale, followed by significantly less monounsaturated fatty acids (MUFA), while several polyunsaturated fatty acids (PUFA) were present in lower but considerable amounts. In all of these PL subclasses, the most abundant fatty acids were the SFA stearic acid (SA; 18:0), followed by the palmitic acid (PA; 16:0) and less amounts of the myristic acid (14:0). From the MUFA, oleic acid (OA; 18:1 c9) was the most abundant, followed by palmitoleic acid (16:1 c9).

All these PL subclasses contained much lower amounts of PUFA, with the n-6 PUFA linoleic acid (LA; 18:2 n-6) being the most abundant PUFA in the SM and PE subclasses, followed by α-linolenic acid (ALA; 18:3 n-3). The PC subclass contained also LA and ALA in similar amounts with the ones observed for these PUFA in both SM and PE subclasses. However, in the PC subclass, the most abundant PUFA was the n-3 PUFA docosahexaenoic acid (DHA; 22:6 n-3), followed by lesser amounts of the n-3 PUFA eicosapentaenoic acid (EPA; 20:5 n-3) and less but detectable amounts of their precursor docosapentaenoic acid (DPA; 22:5 n-3). Notably, EPA and DPA were not detected in SM and only traces of DPA were detected in PE, with these two PL subclasses containing approximately 2–3 orders of magnitude lower amounts of DHA in comparison to those of the PC subclass.

Thus, the n-6 PUFA content in both SM and PE subclasses were higher than their n-3 PUFA content, while the opposite was observed in the PC subclass in which its n-3 PUFA content was significantly higher than its n-6 PUFA content. Subsequently, the levels of the n-6/n-3 PUFA ratio were within the range of approximately 3.5–4.5 for the SM and PE subclasses and much lower for the PC subclass, approximately 0.4.

### 3.3. Structural Elucidation of the Most Bioactive PL Subclasses, SM, PC and PE, from Irish Ale by LC-MS and Structure–Activity Relationships

During the LC-MS analysis, the separation of the lipids was based on the length of the nonpolar acyl- or alkyl-groups in combination with their degree of unsaturation by using a C18 reverse-phase column (characteristic chromatograms of this analysis are shown in the supplementary data, as Figure S2). Moreover, by applying Q-TOF mass spectrometry, simultaneously with the HPLC separation for each one of these peaks, unique MS data were obtained for each peak, leading to complete structural elucidations of novel structures for these ale PC and PE molecules that co-migrated in the TLC band of the SM family. The characterization of these molecules was based on the acquired *m*/*z* values of the demethylated negative ions [M − CH_3_]^−^ for PC and SM molecules of the PC and SM subclass of Irish ale, respectively, while the dehydrogenated negative ions [M − H]^−^ for PE molecules of the PE subclass and for all the other sphingolipids (e.g., ceramides and cerebrosides) and glycolipids (e.g., monogalactosyldiacylglycerol: MGDG) co-migrated at the SM subclass of Irish ale, as previously described for bioactive PL subclasses in other foods [23,24] and further verified by using the LIPID MAPS: Nature Lipidomics Gateway (www.lipidmaps.org) based on the lowest delta values during identification in combination with their fatty acids contents that were acquired by the LC-MS analyses of the FFA derived by the saponification of these ale PL subclasses.

With respect to the LC-MS structural analysis of PC and PE subclasses of Irish ale, survey scans in the negative ion mode between 600 and 1000 *m*/*z* of MS1 demonstrated similar patterns for these subclasses. More specifically, both PC and PE subclasses of Irish ale contain several PC and PE molecules, many of which were diacyl-PC and diacyl-PE molecules, respectively, baring mostly SFA containing 10–20 carbon chains (10:0; 12:0; 14:0; 16:0; 18:0; and 20:0) or the 16:1 MUFA at the *sn*-1 position of their glycerol backbone while, at the *sn*-2 position, either the most abundant SFA (14:0; 16:0; 18:0) or MUFA (16:1; 18:1). Considerable amounts of 1-alkyl-2-*sn*-acyl-PC/PE molecules were also identified in both PC and PE subclasses of Irish ale, with SFA being again the dominant FA moieties at the *sn*-1 position of their glycerol backbone, while at their *sn*-2 position, the most abundant was again either SFA (14:0; 16:0; 18:0) or MUFA (16:1; 18:1; 20:1).

**Figure 2:**
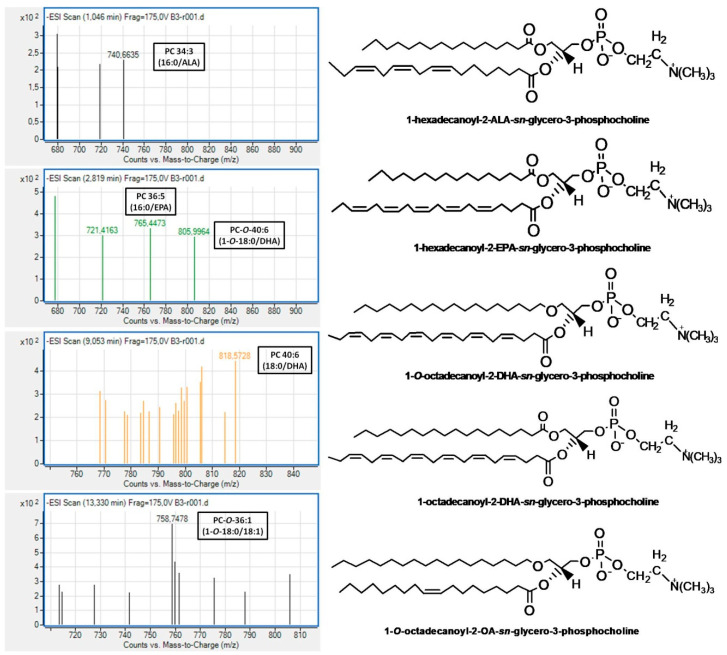
Representative mass spectra and structures of the bioactive PC molecules identified in the PC subclass of Irish ale. PC = phosphatidylcholine; ALA = α-linolenic acid (18:3 n-3); EPA = eicosapentaenoic acid (20:5 n-3); DHA = docosahexaenoic acid (22:6 n-3); and OA = oleic acid.

**Figure 3:**
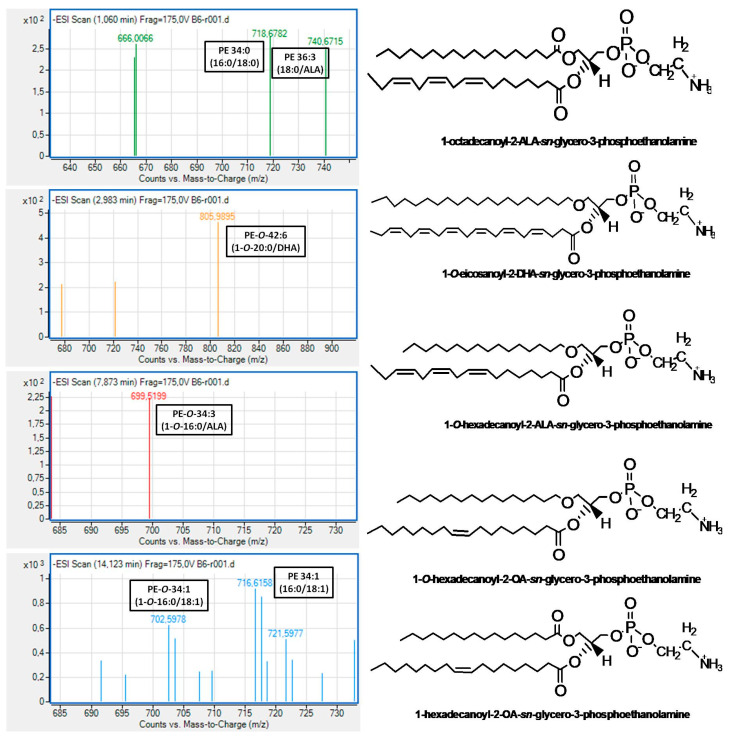
Representative mass spectra and structures of the bioactive PE molecules identified in the PE subclass of Irish ale. PE = Phosphatidylethanolamine; ALA = α-linolenic acid (18:3 n-3); DHA = docosahexaenoic acid (22:6 n-3); and OA = oleic acid.

In addition, in both subclasses, less but considerable amounts of either diacyl or alkyl-acyl bioactive PC and PE molecules bore n-3 PUFA at the *sn*-2 position of their glycerol backbone. In the case of these bioactive PC molecules, the most abundant n-3 PUFA at the *sn*-2 position was the DHA (22:6 n-3) followed by ALA (18:3 n-3) and EPA (20:5 n-3), while in the case of the bioactive PE molecules, the ALA was the most dominant followed by DHA and the n-6 PUFA LA (18:2 n-6). Notably, PE molecules, either diacyl or alkyl-acyl, bearing EPA were not identified in the PE subclass of Irish ale. Moreover, bioactive alkyl-acyl/diacyl PC and PE molecules bearing MUFA (16:1 or 18:1) at the *sn*-2 position of their glycerol backbone were also identified. Representative MS spectra for all these bioactive PC and PE molecules bearing n-3 PUFA or MUFA at their *sn*-2 positions, which were identified in the PC and PE subclasses of Irish ale, are shown in Figure 2 and Figure 3 (All identified PC and PE molecules along with their retention times and m/z values from this analysis are shown in the supplementary data as Tables S1 and S2).

With respect to the LC-MS analysis of the SM subclass, the sphingolipids identified were mainly SM and specific ceramides, such as those with sphinganine (dihydrosphingosine) (e.g., d16:0 abd d18:0), sphingosine (4-sphingenine) (e.g., d16:1 and d18:1), 4,8-sphingodienine (e.g., d18:2), deoxysphinganine (e.g., m18:0) and phytosphingosine (4-hydrosphinganine) (e.g., t18:0 and t18:1), as their base. Several phosphoceramides (e.g., Cer-P: 1-phospho-ceramide), phosphorylethanolamine-ceramides (e.g., PE-Cer: 1-phosphorylethanolamine-ceramide) and hecosylcerebrosides (HexCer: cerebrosides with 1 hexose moiety, either glucose or galactose) were also identified.

The most abundant species were those with SFA or MUFA with 16 or 18 carbons at their fatty chains in the *sn*-1 and *sn*-2 positions, which were mostly eluted after 8 min of this analysis, such as the SM and Cer d36:1 (d18:0/18:1), SM and Cer t36:1 (t18:1/18:0), SM and Cer d32:1 (d16:0/16:1), SM and Cer t32:1 (t16:0/16:1), SM and Cer-P t36:0 (t18:0/18:0), SM and PE-Cer d34:0 (d16:0/18:0), as well as SM and PE Cer-t34:0 (t18:0/16:0). Moreover, less but considerable amounts of sphingolipids bearing the n-3 PUFA ALA (18:3 n-3) were also identified in the bioactive SM subclass of Irish ale, such as the SM d34:3 (d16:0/18:3 n-3); ceramides like the Cer d38:3 (d20:0/18:3 n-3); phosphoceramides like Cer-P d34:3 (d16:0/18:3 n-3), Cer-P d36:3 (d18:0/18:3 n-3) and Cer-P d38:3 (d20:0/18:3 n-3), and phosphorylethanolamine ceramides like the PE-Cer t36:3 (t18:0/18:3 n-3). 

Furthermore, several cerebrosides were also identified, bearing one hexose moiety (glucose or galactose) at their polar head and mostly the 16:0 and 18:0 SFA and the 16:1 and 18:1 MUFA at their fatty chains, while cerebrosides bearing the n-3 PUFA ALA (18:3 n-3) were also identified, such as the HexCer d36:3 (d18:0/18:3 n-3) and the HexCer d32:3 (d14:0/18:3 n-3). In addition, lower but considerable amounts of MGDG were also identified in the SM subclass of Irish ale. Such MGDG molecules had an SFA at the *sn*-1 position and mostly the n-3 PUFA ALA (18:3 n-3) at their *sn*-2 position (all identified sphingolipid and glycolipid molecules, along with their retention times and m/z values from this analysis, are shown in supplementary data as Table S3).

## 4. Discussion

Within the present study, bioactive PL extracts from a specific Irish ale with potent antithrombotic properties against PAF [14] were further fractionated into PL subclasses by TLC, and each TLC derived PL subclass was evaluated for its potential antithrombotic properties by assessing their putative inhibitory effect of PAF-induced aggregation of human platelets while the overall structures and fatty acid composition of the most bioactive PL subclasses of Irish ale PL extracts were elucidated through ESI-LC-MS analysis. Such an experimental approach, using preparative TLC for separating the PL into several subclasses and identifying molecular species of each subclass by ESI-LC-MS, has previously been effectively used in yeasts for beer production [28] and in other food sources [23,24].

More specifically, PL from the specific Irish ale assessed in the present study share similar TLC profiles of PL subclasses with those that were previously derived from TLC analyses of bioactive PL extracts from other types of Irish ale, lager and stout [14] and with other food sources [22,23,24]. Each PL subclass obtained by the TLC analysis of the Irish ale PL extracts was further assessed for its ability to inhibit PAF-induced platelet aggregation in hPRP as previously described [14,23,28]. TLC bands 2 and 3 corresponding to the PL subclasses of the SM family, and PC of Irish ale exhibited the strongest anti-PAF biological activities, followed by the TLC band 5 corresponding to the PE subclass of Irish ale that also showed a strong anti-PAF potency. These results are in accordance with previous studies in the same PL subclasses of other types of Irish ale, larger and stout [14] and of other food sources [23,24] against PAF. In contrast, TLC bands 1 and 6 corresponding to TPL subclasses of lyso-PC and CL displayed poor inhibition against PAF, while some moderate antithrombotic activity was shown by band 4 corresponding to the lyso-PE subclass. Nevertheless, the previously described strong antithrombotic properties of PL extracts derived from the specific Irish ale, with IC_50_ values within the range of approximately 2–10 μg against the PAF pathway of aggregation of human platelets [13], seem to be attributed to the synergism of highly bioactive PL molecules in the PL subclasses of SM, PC and PE that coexist in the specific Irish ale.

The observed strong antithrombotic properties of PL from Irish ale seem to be related to the presence of highly bioactive specific PL molecules in each one of its most bioactive SM, PC and PE subclasses, as previously described in other food sources [23,24]. Therefore, the fatty acid composition and the overall structures of bioactive molecules present in the most bioactive SM, PC and PE subclasses of Irish ale were elucidated through LC-MS analysis as previously described [24].

With respect to the fatty acid profile of all the bioactive PL subclasses (SM, PC and PE) from Irish ale, it was found that they contain mostly SFA, followed by significantly less amounts of MUFA and less but considerable amounts of PUFA. Moreover, in all these highly bioactive PL subclasses, their n-6/n-3 PUFA ratio had favourably low levels that are usually present in healthy foods and diets and were much lower than the much higher levels of 15/1–20/1 for this ratio observed in unhealthy Westernized diets [29]. This result further supports the potential health benefits for these highly bioactive PL subclasses of Irish ale against inflammation-related chronic disorders, including CVD, since it has been reported that high values of this ratio are correlated with a higher risk of CVD and other chronic diseases while, the lower the values of this ratio in a food or diet, the better the anti-inflammatory and antithrombotic health outcomes [29].

Furthermore, the presence of PL molecules in these bioactive PL subclasses containing n-3 PUFAs such as DHA, EPA and ALA also enhance their anti-inflammatory and antithrombotic cardioprotective potency, since such bioactive compounds were found to be the ones with the highest antithrombotic potency against the PAF pathway of platelet aggregation in healthy foods [23,24], while only recently it was found that a standard PC containing n-3 PUFA (DHA) possessed strong antithrombotic properties against PAF, ADP, thrombin and collagen, comparable to that of specific antithrombotic drugs such as aspirin and ginkgolides, with higher specificity against the PAF pathway [30]. Taking this into account, the observed favourable low levels for the n-6/n-3 PUFA ratio in the most bioactive Irish ale PL subclasses, SM, PE and especially in PC, further support their antithrombotic and anti-inflammatory potential against chronic disorders in which the inflammatory PAF-pathway is implicated, such as CVD.

The above obtained outcomes from the fatty acid profile of the bioactive PC, PE and SM subclasses of Irish ale PL were consistent with the overall structural analysis of the bioactive molecules being present in these subclasses. More specifically, both PC and PE subclasses of Irish ale contain several PC and PE molecules, many of which were diacyl-PC and diacyl-PE molecules and less amounts of alkyl-acyl PC and alkyl-acyl-PE, respectively, baring mostly SFA or the 16:1 MUFA at the *sn*-1 position of their glycerol backbone and, at the *sn*-2 position, either the most abundant SFA (16:0 or 18:0) or MUFA (16:1 or 18:1), and less but considerable amounts of such PL baring n-3 PUFA at the *sn*-2 position of their glycerol backbone, such as DHA and ALA for both PC and PE molecules and EPA (20:5 n-3) only for the PC molecules. Notably, the lack of PE molecules baring EPA seems to be related to the lower anti-PAF bioactivity observed in the PE subclass of Irish ale whereas the presence of PC molecules baring these n3PUFA contribute to the higher anti-PAF effects of the PC subclass of Irish ale.

Characteristic examples of bioactive PC and PE molecules with strong antagonistic and agonistic effects against the PAF pathway of human platelet aggregation that were identified in the PC and the PE subclasses of Irish ale (Figure 2 and Figure 3) are the alkyl-acyl 1-*O*-18:0-*sn*-2-DHA-PC eluted at 2.5–3.5 min, the diacyl 18:0-*sn*-2-DHA-PC eluted at 7.4–9.1 min and the diacyl 16:0-*sn*-2-ALA-PE eluted at 1.1 min of the LC-MS analysis of the PC subclass and, for the PE subclass, the 1-*O*-20:0-*sn*-2-DHA-PE eluted at 3.0–5.0 min, the 1-*O*-18:0-*sn*-2-ALA-PE elutesd at 7.8–8.5 min and the diacyl 18:0-*sn*-2-ALA-PE eluted at 1.1 min. Moreover, bioactive alkyl-acyl/diacyl PC and PE molecules bearing MUFA (16:1 or 18:1) at the *sn*-2 position of their glycerol backbone were also identified, for which several anti-inflammatory and cardioprotective bioactivities have also been proposed [31]. It should also be mentioned that PC or PE molecules baring further phospho-groups at the sn-1 or sn-2 positions of the glycerol backbone, usually defined as PC(P) and PE(P), were not identified in the assessed Irish ale PL extracts.

After absorption, such dietary bioactive PL (PC and PE molecules, with n-3 PUFA or MUFA at their *sn*-2 position) are usually delivered smoothly into plasma lipoproteins and, from there, to several blood cells and tissues, including platelets, but also to tissues with accessibility issues, such as the brain. Their amphiphilic nature facilitates their journey within the blood stream and their incorporation into cell membranes and for surpassing the blood-brain barrier. After being transferred to blood cells, including platelets, these bioactive PC and PE molecules interact directly through a strong inhibitory antagonistic or a weak agonistic effect or both effects (in different concentrations) against the PAF pathways of activating cells (including platelet aggregation) because of their structural resemblance to the PAF molecule and thus due to antagonism against the binding of PAF on its receptor [3,23,24].

The identified diacyl 18:0-*sn*-2-DHA-PC in the PC subclass of Irish ale is a classic example of such a dietary bioactive PL, since this molecule when previously purchased as a standard (Avanti Polar Lipids, Alabaster, AL, USA) and tested in human platelets exhibited potent antagonistic (in lower concentrations) and agonistic (in higher concentrations) effects against the PAF pathway of human platelet aggregation [30]. Notably, its antagonistic effects against the PAF pathway were comparable to those for classic PAF antagonists for the PAF-R, such as ginkgolide molecules from the *Ginkgo biloba* tree and other potent anti-platelet compounds like aspirin [30].

Apart from their direct effect on the PAF-R, bioactive PL also beneficially modulates PAF metabolism in several cells, including platelets and leukocytes, and plasma, returning PAF levels and activities to homeostatic ones [3]. Some of these PC and PE molecules can also affect platelet aggregation indirectly due to their susceptibility to the enzyme activity of phospholipase A_2_ and the release of their bioactive MUFA and n-3 PUFA from their *sn*-2 position, which affect several thrombotic and inflammatory intracellular signalling pathways and gene expression [3]. For example, the released n-3 PUFA from such PE and PC molecules can beneficially affect the PAF-induced inflammatory pathways of eicosanoids involved in platelet aggregation and other pro-inflammatory cascades by agonistically inhibiting the cyclooxygenases (COX), which are the basic enzymes involved in eicosanoid synthesis from arachidonic acid [1,3].

Therefore, the beneficial anti-inflammatory properties of such bioactive PC and PE molecules present in Irish ale directly and/or indirectly protect against PAF related inflammatory and thrombotic pathways. This further supports the putative health benefits of dietary bioactive PL, such as those found in Irish ale in the present study, in wine [10,20,21] and in other foods [3,9,18,19,22,23,24]. However, further studies are needed to support such a notion, especially for beer and related brewery by-product-derived PL.

In contrast to the PC and PE subclasses, the LC-MS analysis of the TLC band of the SM subclass of Irish ale revealed that several different classes of bioactive polar lipid molecules were identified, mostly those belonging to the SM family of sphingolipids, such as sphingomyelins, cerebrosides, ceramides and several other glyco-sphingolipids bearing one hexose moiety (glucose or galactose) at their polar head, with various bases at the sphingo-backbone (sphingosine, sphinganine, deoxysphinganine, 4,8-sphingodienine and phytosphingosine), along with several other bioactive polar glycolipids, such as monogalactosyl-diglycerides (MGDG) that co-migrated in the same TLC band. The most abundant molecules in each one of these bioactive lipid subclasses were those with SFA or MUFA with 16 or 18 carbons at their *sn*-1 and *sn*-2 positions, while less but considerable amounts of sphingolipids bearing the n-3 PUFA ALA were also identified.

The identification of several classes of sphingolipids in Irish ale is very important, since several ceramides, cerebrosides and related lipids are metabolites of sphingomyelin and intracellular second messengers, with a wide range of biological activities and health benefits. More specifically, sphingolipids are essential components of the plasma membrane in all eukaryotic cells, including *Saccharomyces cerevisiae* cells. Several sphingolipids are implicated as secondary messengers in vital signalling pathways, and especially in platelets, they play crucial roles in platelet aggregation. There are sphingolipids implicated in platelet aggregation induced by several platelet agonists, such as the sphingosine-1 phosphate molecule [32], while in contrast, others have strong inhibitory effects against platelet aggregation. For example, the SM subclass from several beers and other food sources, such as salmon, strongly inhibit platelet aggregation induced by PAF [14,23,24] and other agonists, such as thrombin, collagen and ADP [23,24]. In addition, specific ceramides identified in cyanobacteria lipid extracts have also exhibited strong anti-PAF effects against platelet aggregation, either by an antagonistic or an agonistic effect against the PAF pathway [33,34].

Sphingolipids are found in many foodstuffs, but the effect of all the classes of dietary sphingolipids on platelets is not fully understood. In addition, the generation of sphingolipids in platelets as second messengers remains elusive, while it is speculated that platelets lack de novo synthesis of ceramides. Therefore, intake of dietary bioactive sphingolipids with strong anti-inflammatory and antithrombotic properties, such as those identified in the SM subclass of Irish ale, salmon and other foods, seems to be of great importance for reducing platelet activation and thus the risk for CVD and other inflammation-related chronic disorders, including cancer [3,16,17].

Apart from their effects in platelets, several types of sphingolipids from beer, beer yeasts and beer-related wastes have exhibited various health benefits against inflammation-related disorders. For example, a specific glycosyl-ceramide (cerebroside) extracted from malt feed of beer brewing waste possess strong antitumour bioactivities [35]. In the present study, in the SM subclass of Irish ale, we identified cerebrosides with similar/identical structures, such as the HexCer-d34:2 (d18:2/16:0). Since PAF is also implicated in cancer and tumour-related metastatic manifestations [3,16,17], it is possible that the antitumour effects of such cerebroside molecules may also be related to their observed strong anti-PAF effects. However, further studies are needed to support such a notion.

Moreover, phytoceramides originating from dietary brewer’s yeast sphingolipids and from relative fermented products such as beer, have been found to be incorporated to several cells, such as hepatocytes and adipocytes, whereby they activate as ligands the peroxisome proliferator-activated receptors (PPARs), regulating the expression of PPARs target genes [36]. As aforementioned, such phytoceramides were also identified in the SM subclass of Irish ale, such as the Cer-t34:2 (t18:1/16:1) molecule. It has been reported that different activations of PPARs by several ligands can promote inflammatory and thrombotic processes [37] or anti-inflammatory and antithrombotic processes against platelet activation and aggregation by other ligands through attenuation of GPCR-related signals [38]. The fact that no platelet aggregatory effects were induced by the SM subclass in the present study suggests that the sphingolipids and glycolipids of this PL subclass of Irish ale possess only anti-PAF effects against platelet aggregation, which seem to be related to their effects on the GPCR PAF-R-related signalling pathways. However, more studies are required to elucidate the exact mechanisms involved in these beneficial effects of the identified glycolipids and sphingolipids of Irish ale.

In addition, the lower but considerable amounts of MGDG identified in the same SM subclass had an SFA at the *sn*-1 position and mostly the n-3 PUFA ALA (18:3 n-3) at their *sn*-2 position, a result that comes in accordance with such glycolipids in beers and cereals for beer production [39]. Such glycolipids have been found to possess several anti-inflammatory and antitumour properties [40]. For example, MGDG rich in ALA has been proposed to reduce platelet aggregation through inhibition of the COX synthesis of eicosanoids and related pathways by an antagonistic effect of ALA against arachidonic acid (ARA) for COX-1 and COX-2. MGDG has also been found to possess anti-inflammatory activity in human articular cartilage too, possibly by an activation of an anti-inflammatory loop triggered by COX-2 for resolving the initial inflammatory signal [41]. In addition, the treatment of avian articular chondrocytes with MGDG suppressed the expression of inflammation-induced proteins, suggesting a strong anti-inflammatory property of MGDG [42]. Nevertheless, more studies are required for the evaluation of the bioactivities of several glycolipid molecules in beers and brewery by-products against the PAF pathway, inflammation, thrombosis and related chronic disorders.

## 5. Conclusions

This is the first study to elucidate the structures of bioactive PL molecules of Irish ale that have potent anti-inflammatory and antithrombotic properties against the PAF pathway. Several bioactive diacyl and alkyl-acyl PC molecules containing n-3 PUFA, mostly DHA followed by ALA and EPA, and MUFA such as OA were identified in the PL of Irish ale. In addition, similar bioactive diacyl and alkyl-acyl PE molecules bearing n-3 PUFA, mostly ALA followed by DHA, and MUFA such as OA were also identified. The lower amount of PE molecules bearing DHA and the lack of PE molecules bearing EPA may explain the lower bioactivities found in PE against PAF compared to the higher anti-PAF potency found in PC molecules, in which much higher amounts of DHA and EPA were present. In addition, several sphingolipids and glycolipids with a wide range of bioactivities and health benefits were also identified in the SM subclass of Irish ale. Furthermore, in all the bioactive PL subclasses and especially in the PC subclass, favourable low levels of the n-6/n-3 PUFA ratio were observed, which further support their anti-inflammatory potential against chronic disorders.

The novel results of the present study further support the valorisation of bioactive PL from beer and brewery by-products with strong antithrombotic properties against the PAF-related inflammatory pathways and associated chronic disorders, such as CVD, cancer and neurodegenerative disorders.

## 6. Limitations—Future Perspectives

The results of the present study on the antithrombotic properties of the Irish ale bioactive PL extracts and identified subclasses and molecules are based on an in vitro experimental design. More ex vivo and in vivo studies are required to further support the observed bioactivities and structure activity relationships of the antithrombotic Irish ale PL. For example, studies investigating the potential ex vivo antithrombotic postprandial effect of Irish ale and/or in vivo interventions for evaluating the beneficial long-term effect of moderate consumption of Irish ale, its by-products or its bioactive constituents in healthy subjects versus patients with inflammation and thrombosis-related disorders will provide additional evidence of health benefits of such bio-functional fermented beverages.

## Figures and Tables

**Table 1:** The fatty acid profile of the most bioactive PL subclasses of Irish ale PL.

[M − H]^−^	Fatty Acid	B2 (SM)	B3 (PC)	B5 (PE)
143.108	8:0	0.003 ± 0.001	0.008 ± 0.002	0.004 ± 0.004
157.123	9:0	0.021 ± 0.001 *	0.007 ± 0.002	0.011 ± 0.003
171.139	10:0	0.007 ± 0.001	0.010 ± 0.002	0.016 ± 0.001
185.155	11:0	0.011 ± 0.001	0.012 ± 0.003	0.015 ± 0.002
199.170	12:0	0.564 ± 0.025	0.375 ± 0.032	0.704 ± 0.052 *
213.186	13:0	0.072 ± 0.002 *	0.042 ± 0.003	0.059 ± 0.005
227.202	14:0	4.298 ± 0225 *	2.726 ± 0222	2.822 ± 0205
241.217	15:0	0.450 ± 0.038	0.252 ± 0.027	0.260 ± 0.010
255.233	16:0 (PA)	27.765 ± 4.121	33.628 ± 3.633	27.899 ± 4.063
253.217	16:1 c9	1.811 ± 0.337 *	0.567 ± 0.049	0.555 ± 0.042
269.249	17:0	1.909 ± 0.215 *	1.207 ± 0.226	1.627 ± 0.042
283.264	18:0	42.908 ± 7.256	41.338 ± 2.017	48.060 ± 6.425
281.249	18:1 c9 (OA)	17.580 ± 2.050	14.944 ± 1.309	15.744 ± 2.317
279.233	18:2 c9, c12 (LA)	1.030 ± 0.163	0.874 ± 0.100	0.741 ± 0.026
307.264	18:2 c10, c12	0.117 ± 0.009	0.098 ± 0.019	0.079 ± 0.003
277.217	18:3 c9, c12, c15 (ALA)	0.287 ± 0.023	0.245 ± 0.019	0.164 ± 0.011
275.202	18:4 c6, c9, c12, c15	ND	0.020 ± 0.001	ND
297.280	19:0	0.168 ± 0.026	0.123 ± 0.016	0.163 ± 0.004
311.296	20:0	0.131 ± 0.009	0.133 ± 0.010	0.139 ± 0.008
309.280	20:1 c9	0.421 ± 0.094	0.475 ± 0.143	0.470 ± 0.020
305.249	20:3 c8, c11, c14	0.003 ± 0.002	0.019 ± 0.002	0.009 ± 0.001
303.233	20:4 c5, c8, c11, c14 (ARA)	0.005 ± 0.005	0.033 ± 0.009 *	0.008 ± 0.001
301.217	20:5 c5, c8, c11, c14, c17 (EPA)	ND	0.353 ± 0.309	ND
325.311	21:0	0.005 ± 0.007	0.009 ± 0.008	0.015 ± 0.013
335.296	22:2 c13, c16	ND	0.013 ± 0.011	0.013 ± 0.011
333.280	22:3 c5, c13, c16	0.035 ± 0.003	0.020 ± 0.003	0.024 ± 0.002
329.249	22:5 c7, c10, c13, c16, c19 (DPA)	ND	0.048 ± 0.013 *	0.001 ± 0.001
327.233	22:6 c4, c7, c10, c13, c16, c19 (DHA)	0.009 ± 0.008	1.818 ± 0.174 *	0.039 ± 0.003
353.343	23:0	0.044 ± 0.076	0.038 ± 0.066	0.014 ± 0.013
367.358	24:0	0.349 ± 0.605	0.565 ± 0.611	0.345 ± 0.327
	SFA	78.703 ± 0.788	80.473 ± 0.430	82.152 ± 0,699
	MUFA (n9)	19.812 ± 0.827	15.986 ± 0.500 *	16.769 ± 0.793
	PUFA	1.485 ± 0.031	3.528 ± 0.065 *	1.066 ± 0.006
	n-3	0.296 ± 0.016	2.485 ± 0.103 *	0.205 ± 0.005
	n-6	1.189 ± 0.037 *	1.056 ± 0.024	0.874 ± 0.007
	n-6/n-3	4.021 ± 0.335	0.425 ± 0.027 *	4.268 ± 0.140

Results are expressed as a percentage of each fatty acid on the total fatty acid content of each sample (mean ± SD, n = 3). The asterisk (*) indicates statistically significant differences observed when fatty acid compositions of the same row are compared using Kruskal Wallis nonparametric multiple comparison test (*p* ≤ 0.05). n-6/n-3 ratio uncertainty was calculated using the following equation: ∆x/x = ([∆n]6/n-6 + [∆n]3/n-3) × n-6/n-3. Abbreviations: B2 (SM), B3 (PC) and B5 (PE) represent the TLC band containing the PL subclasses of SM, PC and PE; c = cis; SFA = saturated fatty acids; MUFA = monounsaturated fatty acids; PUFA = polyunsaturated fatty acids; n-6 = omega-6; n-3 = omega-3; ALA = α-linolenic acid; DHA = docosahexaenoic acid; EPA = eicosapentaenoic acid; DPA = docosapentaenoic acid; ARA = arachidonic acid; OA = oleic acid; and ND: non-detectable.

## Supplementary Materials

The following are available online at https://www.mdpi.com/2218-273X/10/7/1075/s1, Figure S1: Representative chromatograms of the LC-MS analysis of the saponified (free fatty acids) of the most bioactive PL subclasses (TLC bands B2, B3 and B5, respectively) of Irish ale PL., Figure S2: Representative chromatograms of the LC-MS analysis of the unsaponified bioactive PL subclasses (TLC bands B2, B3, and B5, respectively) of Irish ale PL, Table S1: Identified molecules of the bioactive PC subclass of Irish ale, Table S2: Identified molecules of the bioactive PE subclass of Irish ale, Table S3: Identified sphingolipid and glycolipid molecules of the bioactive SM subclass of Irish ale.
