# Supplementary material for: Structural Elucidation of Irish Ale Bioactive Polar Lipids with Antithrombotic Properties
Source: Biomolecules. 2020 Jul 18;10(7):1075. doi: 10.3390/biom10071075 (PMC7407377; doi:10.3390/biom10071075)
Supplement: Supplementary file 1 [file biomolecules-10-01075-s001.pdf]

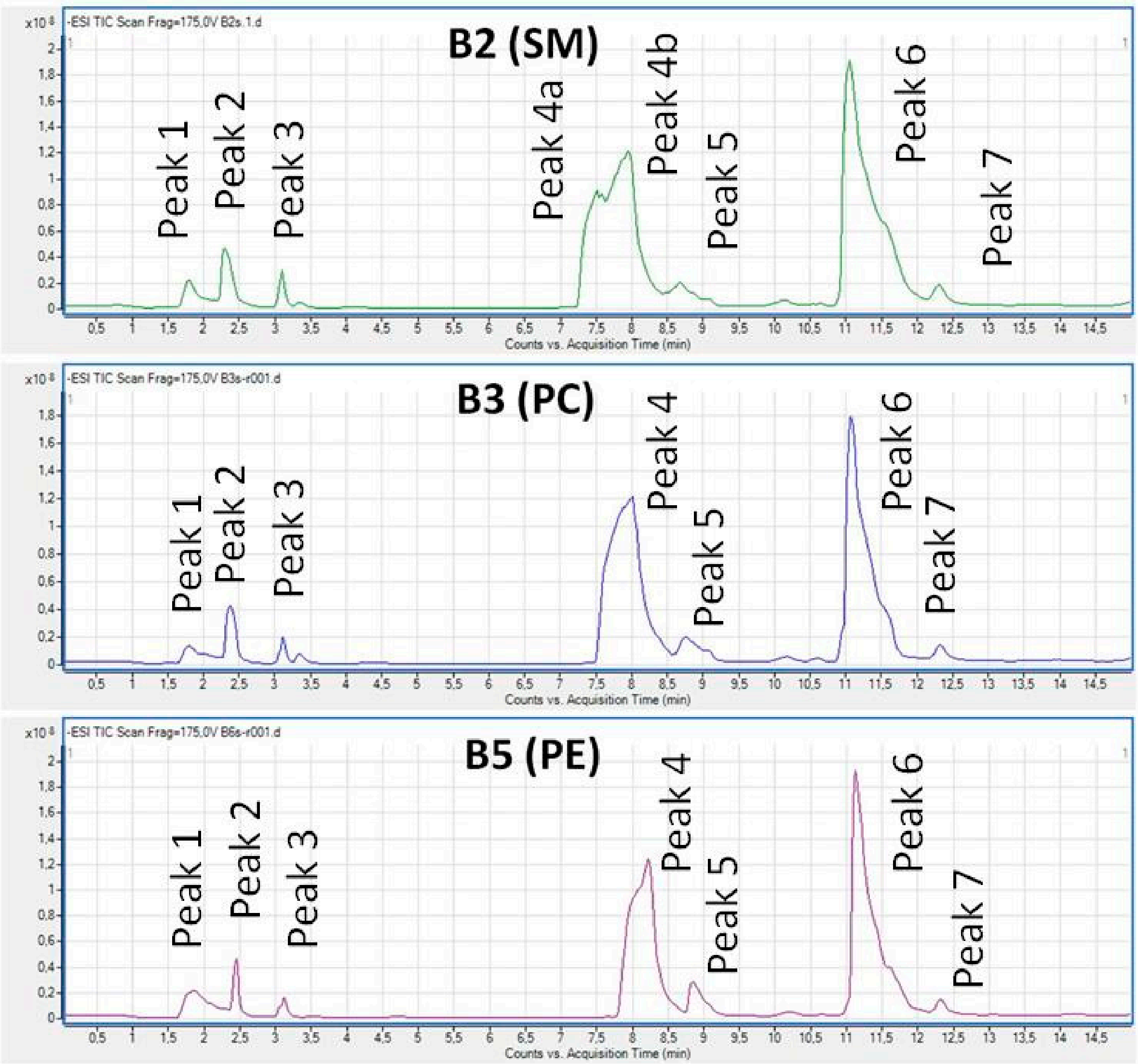

Figure S1. Representative chromatograms of the LC-MS analysis of the saponified (free fatty acids) of the most bioactive PL subclasses (TLC bands B2, B3 and B5, respectively) of Irish ale PL. Abbreviations: B2 (SM), B3 (PC) and B5 (PE) represent the TLC bands containing the PL subclasses of the sphingomyelin family (SM), phosphatidylcholines (PC) and phosphatidylethanolamines (PE), respectively.

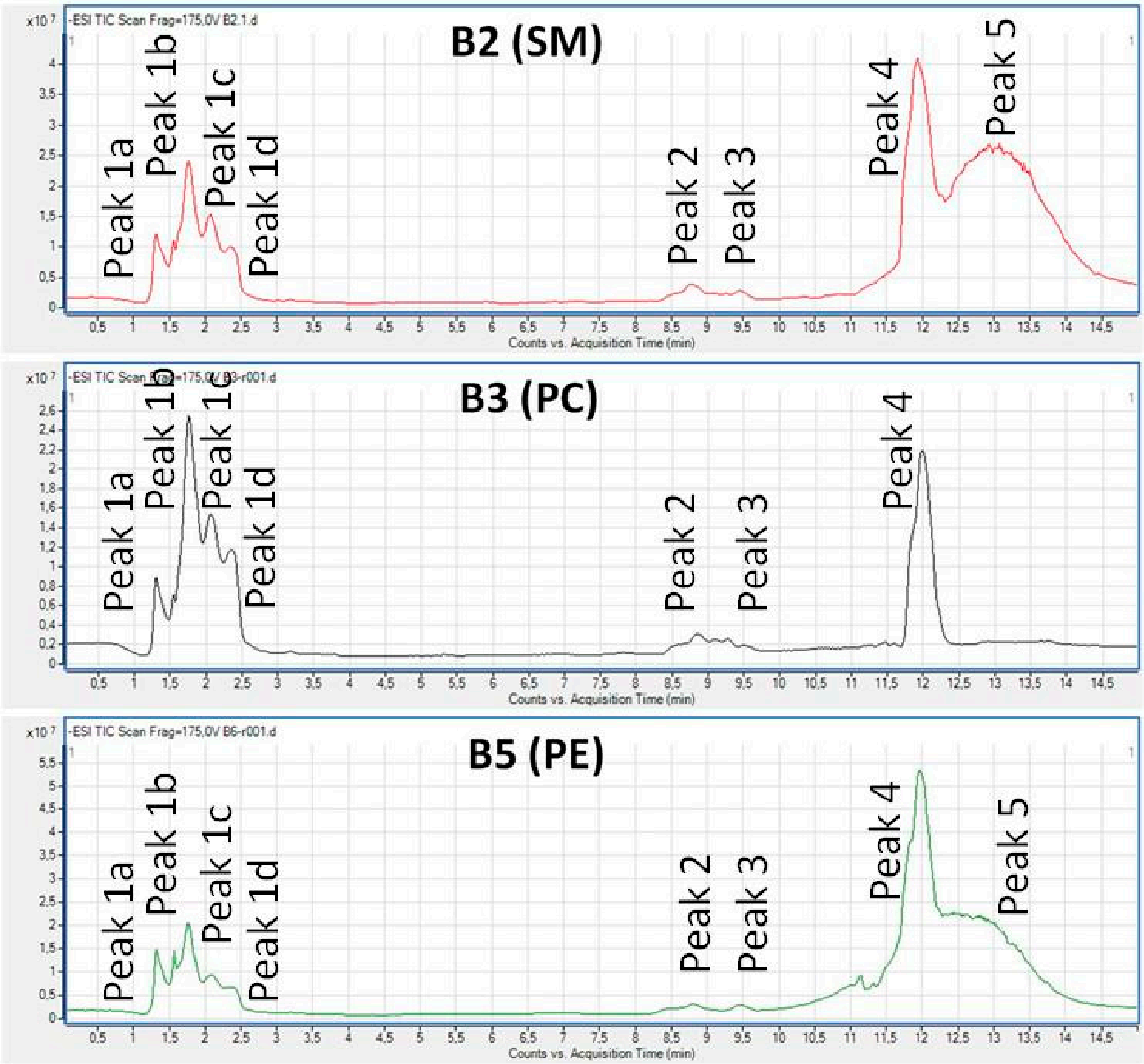

Figure S2. Representative chromatograms of the LC-MS analysis of the unsaponified bioactive PL subclasses (TLC bands B2, B3, and B5, respectively) of Irish ale PL. Abbreviations: B2 (SM), B3 (PC), and B5 (PE) represent the TLC bands containing the PL subclasses of the sphingomyelin family (SM), phosphatidylcholines (PC), and phosphatidylethanolamines (PE), respectively.

Table S1. Identified molecules of the bioactive PC subclass of Irish ale.

Elution time (min)	[M-CH_3]$^-$	Identified molecular species of the bioactive ale PC subclass
1.046	740.6635	PC 34:3 (16:0/18:3 n-3) *
1.095	680.0449	PC 30:5 (10:0/20:5 n-3)
1.775	648.4354	PC-*O*-28:0 (1-*O*-14:0/14:0 or 1-*O*-12:0/16:0)
2.405	691.4650	PC 30:0 (14:0/16:0 or 12:0/18:0)
2.554 – 4.447	805.9872	PC-*O*-40:6 (1-*O*-18:0/22:6 n-3)
2.753	677.3832	PC-*O*-30:0 (1-*O*-14:0/16:0 1-*O*-12:0/18:0)
2.819	765.4473	PC 36:5 (16:0/20:5 n-3)
3.021 – 3.330	716.7208	PC 32:1 (16:0/16:1)
3.052	743.4548	PC 34:2 (16:0/18:2 n-6 or 16:1/18:1)
3.665	824.1209	PC 40:3 (18:0/22:3 n-6 or 22:0/18:3 n-3)
5.057	868.5205	PC-*O*-40:4 (1-*O*-20:0/20:4 n-6)
5.091 - 6.168	734.0158	PC 34:6 (12:0/22:6 n-3)
5.522	733.9848	PC-*O*-34:0 (1-*O*-16:0/18:0 or 1-*O*-18:0/16:0)
5.704	860.8957	PC 43:6 (21:0/22:6 n-3)
6.516	718.4787	PC 32:0 (14:0/18:0 or 18:0/14:0 or 16:0/16:0)
6.599 – 8.290	685.4834	PC 30:2 (12:0/18:2 n-6)
6.715 - 6.782	700.4945	PC-*O*-32:2 (1-*O*-16:1/16:1 or 1-*O*-14:0/18:2 n-6)
6.782	773.2687	PC 36:1 (18:0/18:1)
7.130 - 7.975	702.4938	PC-*O*-32:1 (1-*O*-16:0/16:1)
7.412 – 9.103	818.5905	PC 40:6 (18:0/22:6 n-3)
8.290 – 9.103	769.4212	PC 36:3 (18:0/18:3 n-3)
9.103	726.5390	PC-*O*-34:3 (1-*O*-16:0/18:3 n-3)
	879.7304	PC 44:4 (24:0/20:4 n-6)
9.252	798.5939	PC 38:2 (20:0/18:2 n-6)
9.368 – 9.517	737.4968	PC 34:5 (14:0/20:5 n-3)
9.517	784.5858	PC-*O*-38:2 (1-*O*-20:0/18:2 n-6 or 1-*O*-18:1/20:1)
10.876	714.6055	PC 32:2 (16:1/16:1 or 14:0/18:2 n-6)
11.800 – 12.400	786,7672	PC-*O*-38:1 (1-*O*-18:0/20:1)
11.987 – 12.219	876.7725	PC 44:5 (24:0/20:5 n-3)
12.451 – 14.789	775.6044	PC 36:0 (18:0/18:0)
8.671-13.500	758.7404	PC-*O*-36:1 (1-*O*-18:0/18:1)
14.606	705.5763	PC-*O*-32:0 (1-*O*-16:0/16:0 or 1-*O*-14:0/18:0)
14.789	707.5792	PC 32:6 (10:0/22:6 n-3)
14.838	727.5560	PC-*O*-34:3 (1-*O*-16:0/18:3 n-3)

* indicates the most probable structures with specific fatty acids at the *sn*-1/*sn*-2 positions for the identified molecule of the bioactive PC subclass of Irish ale verified by using the LIPID MAPS: Nature Lipidomics Gateway (www.lipidmaps.org), based on the lowest delta values during identification, in combination with their fatty acids contents that were acquired by the LC-MS analyses of the FFA

derived by the saponification of these ale PL subclasses (Table 2). PC = phosphatidylcholine; n-3: omega-3 PUFA; n-6: omega-6 PUFA.

Table S2. Identified molecules of the bioactive PE subclass of Irish ale

Elution time (min)	[M-H]⁻	Identified molecular species of the bioactive ale PE subclass
0.911	705.5685	PE-*O*-34:0 (1-*O*-16:0/18:0)
1.060	718.6782	PE 34:0 (16:0/18:0)
1.060 - 1.150	740.6590	PE 36:3 (18:0/18:3 n-3)
1.110	620.7077	PE-*O*-28:0 (1-*O*-12:0/16:0 or 1-*O*-14:0/14:0)
1.226 - 1.400	683.4323	PE 32:4 (12:0/20:4 n-6)
1.226 – 1.491	725.5039	PE-*O*-36:4 (1-*O*-18:1/18:3 n-3)
	739.5114	PE 36:4 (18:1/18:3 n-3)
	697.4492	PE-*O*-34:4 (1-*O*-16:1/18:3 n-3)
	711.4630	PE 34:4 (16:1/18:3 n-3)
1.756	648.4306	PE-*O*-30:0 (1-*O*-14:0/16:0 or 1-*O*-12:0/18:0)
2.801	724.4076	PE 35:6 (13:0/22:6 n-3)
3.033	729.4428	PE-*O*-36:2 (1-*O*-18:0/18:2 n-6 or 1-*O*-18:1/18:1)
	743.4602	PE 36:2 (18:0/18:2 n-6 or 18:1/18:1)
3.265	733.6236	PE 35:0 (17:0/18:0)
2.985- 5.188	805.9834	PE-*O*-42:6 (1-*O*-20:0/22:6 n-3)
3.795	736.7255	PE 36:5 (18:2/18:3 n-3)
4.027	838.2827	PE-*O*-44:3 (1-*O*-24:0/20:3 n-6)
5.337	853.9842	PE 44:3 (24:0/20:3 n-6)
6.229 – 10.440	734.0084	PE 36:6 (18:3 n-3/18:3 n-3 or 14:0/22:6 n-3)
6,846 – 8.835	686,4898	PE 32:2 (16:1/16:1 or 14:0/18:2 n-6)
6,680 – 8.686	675,4654	PE-*O*-32:1 (1-*O*-16:0/16:1 or 1-*O*-14:0/18:1)
7.227 – 8.686	677.4710	PE-*O*-32:0 (1-*O*-16:0/16:0 or 1-*O*-14:0/18:0)
7.608	685.5027	PE 32:3 (16:1/18:2 or 14:0/18:3 n-3)
7.757	655.4299	PE 30:4 (10:0/20:4 n-6)
7.807 – 8.454	699.4985	PE-*O*-34:3 (1-*O*-16:0/18:3 n-3)
8.072	715.1433	PE 34:2 (16:1/18:1 or 16:0/18:2 n-6)
8.338	691.3995	PE 32:0 (16:0/16:0 or 14:0/18:0)
8.835	658.4737	PE 30:2 (12:0/18:2 n-6)
9.415	786.7701	PE 40:8 (18:2n6/22:6 n-3) or PE-*O*-40:1 (1-*O*-20:0/20:1)
9.962 – 12.731	788.7791	PE 40:7 (18:1/22:6 n-3) or PE-*O*-40:0 (1-*O*-20:0/20:0)
10.891	732.7244	PE-*O*-36:0 (1-*O*-18:0/18:0)
11.073	679.5533	PE 32:6 (10:0/22:6 n-3)
11.106	872.8778	PE-*O*-46:0 (1-*O*-23:0/23:0)
11.885 – 12.731	816.8134	PE 42:7 (20:1/22:6 n-3) or PE-*O*-42:0 (1-*O*-18:0/24:0)
11.918	814.7911	PE-*O*-42:1 (1-*O*-24:0/18:1)
12.416 - 12.731	760.7477	PE 38:7 (16:1/22:6 n-3) or PE-*O*-38:0 (1-*O*-18:0/20:0)
13.576 – 14.123	716.6140	PE 34:1 (16:0/18:1 or 18:0/16:1)

13.891 – 14.123	702.6001	PE-*O*-34:1 (1-*O*-16:0/18:1 or 1-*O*-18:0/16:1)
14.123	721.5977	PE 35:6 (13:0/22:6 n-3)

* indicates the most probable structure with specific fatty acids at the *sn*-1/*sn*-2 positions for the identified molecule of the bioactive PE subclass of Irish ale verified by using the LIPID MAPS: Nature Lipidomics Gateway (www.lipidmaps.org), based on the lowest delta values during identification, in combination with their fatty acids contents that were acquired by the LC-MS analyses of the FFA derived by the saponification of these ale PL subclasses (Table 2). PE = phosphatidylethanolamine; n-3: omega-3 PUFA; n-6: omega-6 PUFA.

Table S3. Identified sphingolipid and glycolipid molecules of the bioactive SM subclass of Irish ale

Elution time (min)	[M-CH_3]$^-$	Identified SM molecular species of the bioactive ale SM-subclass	Elution time (min)	[M-H]$^-$	Identified molecular species of other sphingolipids and glycolipids (ceramides, cerebrosides, MGDG) of the bioactive ale SM subclass
1.787	648.4312	SM t30:1 (t16:1/14:0 or t18:1/12:0)	0.826	524.5329	Cer d36:1 (d18:0/18:1 or d18:1/18:0)*
2.682	633.3638	SM d30:0 (d16:0/14:0 or d18:0/12:0)		613.4985	Cer-P d34:3 (d16:0/18:3 n-3 or d18:2/16:1 or d16:1/18:2 n-6)
2.682 – 3.180	743.6082	SM d38:1 (d18:1/20:0 or d18:0/20:1)		723.5226	HexCer d36:3 (d18:0/18:3 n-3 or d18:2/18:1 or d18:1/18:2 n-6)
2.682 – 3.379	677,3905	SM t32:0 (t16:0/16:0)	0.991	478.7963	Cer d30:2 (d12:0/18:2 n-6 or d18:2/12:0)
5.186 -6.031	733.9905	SM t36:0 (t18:0/18:0)		620.7210	PE-Cer t30:1 (PE-Cer-t14:0/16:1) or Cer d40:1 (d20:0/20:1)
6.223-9.711	684.5017	SM d34:3 (d16:0/18:3 n-3)	1.058	642.6901	Cer-P d36:2 (d18:0/18:2 n-6 or d18:2/18:0 or d18:1/18:1)
6.562 – 8.584	658.4590	SM d32:2 (d14:0/18:2n6 or d18:2/14:0)	1.141	454.3910	Cer d28:0 (d14:0/14:0 or d16:0/12:0)
	702.5010	SM t34:2 (t16:0/18:2n6 or t18:1/16:1)	1.174	438.7930	Cer d26:1 (d16:1/10:0)
6.562 – 7.574	746.5021	SM d38:0 (d18:0/20:0)	1.174 - 1.572	526.3475	Cer t32:0 (t16:0/16:0)
6.794	718.4928	SM d36:0 (d18:0/18:0)	1.373	669.4201	HexCer d32:2 (d16:1/16:1 or d18:2/14:0)
6.794 – 8.020	631.4572	SM d30:1 (d16:1/14:0 or d18:1/12:0)	1.787	648.4312	PE-Cer t32:1 (t16:0/16:1 or t16:1/16:0)
6.794 – 9.612	685.4959	SM d34:2 (d18:1/16:1 or d18:2/16:0)	2.185	723.5155	MGDG 32:3 (14:0/18:3 n-3)
6.993 – 10.225	659.4669	SM d32:1 (d16:1/16:0 or d18:1/14:0)	2.334	525.3883	Cer t32:1 (t16:0/16:1)
7.374 – 8.020	675.4751	SM t32:1 (t16:1/16:0 or t16:0/16:1)	2.682	589.3377	Cer d38:3 (d20:0/18:3 n-3)
10.822-14.668	760.7568	SM t38:1 (t18:1/20:0 or t18:0/20:1)		633.3638	Cer-P d34:1 (d16:0/18:1 or d18:0/16:1 or d18:1/16:0)
10.822–11.402	673.5050	SM t32:2 (t14:0/18:2 n-6)	3.180	641.4270	Cer-P d36:3 (d18:0/18:3 n-3 or d18:1/18:2 n-6 or d18:2/18:1)
11.402–12.065	699.5221	SM d35:2 (d18:2/17:0)	3.379	606.4806	Cer-P t32:0 (t16:0/16:0)
11.402–14.668	788.7843	SM t40:1 (t20: 0/20:1)	4.838	666.0056	HexCer d32:3 (HexCer d14:0/18:3 n-3)

11.552–12.065	649.5116	SM t30:0 (t16:0/14:0 or t18:0/12:0)
12.248-13.425	662.6401	SM d32:0 (d16:0/16:0)
	732.7234	SM t36:1 (t18:1/18:0 or t18:0/18:1)
14.668–14.817	705.5701	SM t34:0 (t16:0/18:0 or t18:0/16:0)

5.484	734.0008	Cer-P d38:3 (d20:0/18:3 n-3)
6.412 – 7.258	673.4374	HexCer d32:0 (d16:0/16:0) or Cer-P d38:1 (d20:0/18:1 or d18:1/20:0)
6.526	702.5010	PE-Cer t36:2 (t18:0/18:2 n-6 or t18:1/18:1)
6.794	685.4959	PE-Cer t36:3 (t18:0/18:3 n-3 or t18:1/18:2 n-6)
6.926	554.3555	Cer t34:0 (Cer t16:0/18:0)
6.926 – 7.589	657.4623	PE-Cer d36:2 (d18:0/18:2 n-6 or d18:1/18:1 or d18:2/18:0)
6.993 – 8.810	659.4669	PE-Cer d34:1 (d18:0/16:1 or d18:1/16:0) or Cer-P t36:2 (t18:1/18:1)
6.993	746.5172	PE-Cer d40:0 (d20:0/20:0)
7.109 – 7.954	686.4959	HexCer t32:1 (t16:0/16:1 or t16:1/16:0)
8.020	675.3882	Cer-P d38:0 (d20:0/18:0) or PE-Cer d34:1 (d16:0/18:1 or d18:1/16:0)
8.435	524.5071	Cer t32:1 (t16:0/16:1)
8.584 – 8.853	511.4693	Cer d32:0 (d16:0/16:0)
8.833 – 10.491	550.5136	Cer t34:2 (t18:1/16:1) or Cer-m36:0 (m18:0/18:0)
9.347	579.3945	Cer t36:2 (t18:0/18:2 n-6 or t18:1/18:1) or Cer m36:0 (m18:0/20:0)
9.977 – 13.425	564.5372	Cer d36:1 (d18:0/18:1 or d18:1/18:0)
9.977	623.4899	Cer d40:0 (d20:0/20:0)
10.225–10.491	697.5056	HexCer d34:2 (d18:2/16:0)
10.225	836.5829	MGDG 40:3 (20:0/18:3 n-3)
10.491	609.4724	Cer t38:1 (t20:0/18:1 or t18:0/20:1 or t18:1/20:0)
11.253	673.5054	HexCer t32:0 (d16:0/16:0)
	760.7569	PE-Cer t40:1 (t20:0/20:1)

11.402	699.5221	HexCer d34:1 (d16:0/18:1 or d18:1/16:0) or PE-Cer t36:3 (t18:0/18:3 n-3)
11.552	649.5116	PE-Cer t32:0 (t16:0/16:0)
12.364-12.646	662.6496	Cer-P t36:0 (t18:0/18:0) or PE-Cer d34:0 (d16:0/18:0)
12.646–12.828	509.3868	Cer d32:1 (d16:0/16:1 or d16:1/16:0)
12.828–13.060	671.4873	HexCer d32:1 (d16:0/16:1 or d16:1/16:0) or PE-Cer t34:3 (t16:0/18:3 n-3)
13.425–13.823	677.5294	PE-Cer t34:0 (t18:0/16:0)

* indicates the most probable structure with specific fatty acids at the *sn*-1/*sn*-2 positions for the identified molecule of the bioactive SM subclass of Irish ale verified by using the LIPID MAPS: Nature Lipidomics Gateway (www.lipidmaps.org), based on the lowest delta values during identification, in combination with their fatty acids contents that were acquired by the LC-MS analyses of the FFA derived by the saponification of these ale-PL subclasses (Table 2). SM = sphingomyeline; Cer = ceramides; d = SM or ceramides with a sphinganine (dihydrosphingosine) base when saturated or sphingosine (4-sphingenine) or 4,8-sphingodienine base; m = ceramides with a deoxysphinganine base; t = SM or ceramides with a phytosphingosine (4-hydrosphinganine) base; Cer-P = 1-phospho-ceramide; PE-Cer = 1-phosphorylethanolamine-ceramide; HexCer = cerebrosides with 1 hexose moiety (glucose/galactose); n-3 = omega-3 PUFA; n-6 = omega-6 PUFA.
